# The Aryl Hydrocarbon Receptor in Asthma: Friend or Foe?

**DOI:** 10.3390/ijms21228797

**Published:** 2020-11-20

**Authors:** Odile Poulain-Godefroy, Mélodie Bouté, Julie Carrard, Daniel Alvarez-Simon, Anne Tsicopoulos, Patricia de Nadai

**Affiliations:** University of Lille, CNRS, Inserm, CHU Lille, Institut Pasteur de Lille, U1019-UMR9017-CIIL-Centre d′Infection et d′Immunité de Lille, F59000 Lille, France; odile.poulain@pasteur-lille.fr (O.P.-G.); melodie.boute@outlook.fr (M.B.); carrard.julie@orange.fr (J.C.); danielalvarezsimon@gmail.com (D.A.-S.); patricia.de-nadai@pasteur-lille.fr (P.d.N.)

**Keywords:** asthma, aryl hydrocarbon receptor, immunity

## Abstract

The aryl hydrocarbon receptor (AhR) is a ligand-activated transcription factor that has emerged as an important player in asthma control. AhR is responsive to environmental molecules and endogenous or dietary metabolites and regulates innate and adaptive immune responses. Binding of this receptor by different ligands has led to seemingly opposite responses in different asthma models. In this review, we present two sides of the same coin, with the beneficial and deleterious roles of AhR evaluated using known endogenous or exogenous ligands, deficient mice or antagonists. On one hand, AhR has an anti-inflammatory role since its activation in dendritic cells blocks the generation of pro-inflammatory T cells or shifts macrophages toward an anti-inflammatory M2 phenotype. On the other hand, AhR activation by particle-associated polycyclic aromatic hydrocarbons from the environment is pro-inflammatory, inducing mucus hypersecretion, airway remodelling, dysregulation of antigen presenting cells and exacerbates asthma features. Data concerning the role of AhR in cells from asthmatic patients are also reviewed, since AhR could represent a potential target for therapeutic immunomodulation.

## 1. Introduction

Asthma is an inflammatory disorder of the airways leading to bronchial hyperreactivity and airway obstruction. In this context, the lung barrier surface is crucial to maintain the integrity of the host following exposure to different events. Lung epithelial cells express receptors that detect environmental stimuli, secrete danger signals, and orchestrate defence against inhaled microorganisms, allergens and pollutants. In asthma, the epithelial barrier integrity is compromised, as the epithelium fails to appropriately repair. Furthermore, there is an epithelial immune deviation in mediator production, resulting in inappropriate recruitment and skewing of downstream adaptive immune responses [1]. The mechanisms linking environment and immunity are still not completely understood but may involve environmental sensors. The aryl hydrocarbon receptor (AhR) is a conserved ligand-activated transcription factor highly expressed in the lungs from both humans and mice [2,3]. AhR is a sensor of environmental pollutants, diet molecules, microbial components and metabolic signals that controls complex transcriptional programmes which are ligand- and cell-type- specific. In the last few years, a large body of evidence has shown that AhR is involved in maintaining homeostasis or in eliciting pathology by modulating the biological responses of critical cell types at the barrier and mucosal interfaces. In this review, we summarize our current knowledge about AhR and the cell programmes it controls related to asthma pathophysiology.

### 1.1. Mechanisms of AhR Activation

**Figure 1:**
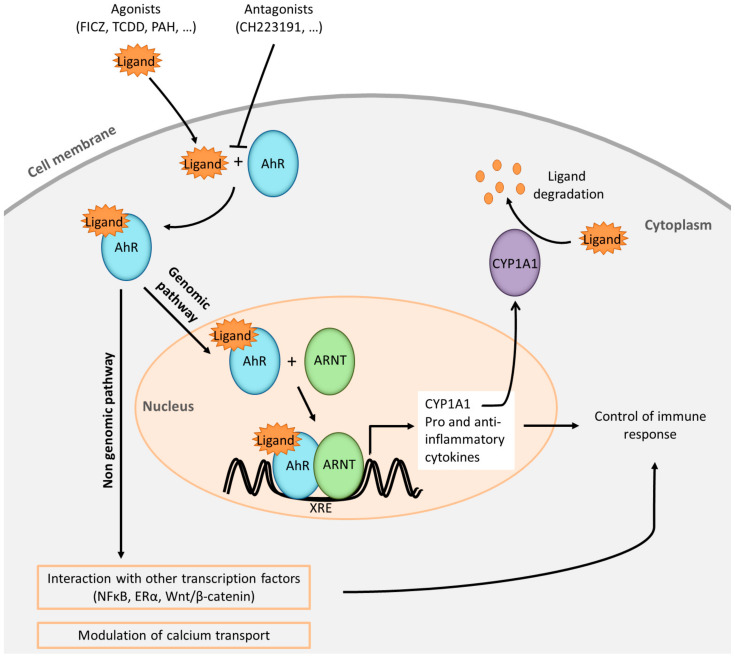
Simplified representation of aryl hydrocarbon receptor (AhR) pathways. AhR ligands (FICZ: 6-formylindolo [3,2-b]carbazole; TCDD: 2, 3, 7, 8-tetrachlorodibenzo-p-dioxin; PAH: polycyclic aromatic hydrocarbons) cross the plasmatic membrane and bind to AhR present in the cytoplasm. The presence of antagonists in the medium is susceptible to block the interaction with the agonists. The ligand-receptor complex translocates into the nucleus and the complex heterodimerizes with its partner ARNT (AhR Nuclear Translocator). The heterodimer binds specific DNA sequences located in the promoter regions of target genes known as xenobiotic response elements (XRE). Among target genes, *CYP1A1* (Cytochrome P450, family 1, subfamily A, polypeptide 1) is a hallmark of AhR signalling. CYP1A1 protein is a member of the cytochrome P450 superfamily of enzymes which one of their roles is to degrade the AhR natural ligands. Numerous genes coding for pro-inflammatory and anti-inflammatory products are induced by this mechanism, which is referred to as the AhR genomic pathway. Non-genomic pathways are also described such as interactions of AhR with other transcription factors or modulation of calcium transport. Altogether these AhR pathways are able to control positively or negatively the immune response.

Upon ligand binding, AhR functions as a transcription factor that affects gene expression via translocation to the nucleus, promoter binding, recruitment of co-activators and co-repressors at specific DNA regions (harbouring or not AhR-responsive DNA elements also known as xenobiotic response elements (XRE)), and via interactions with signal transduction machinery in mouse and human cells. Detailed AhR signalling pathways have been extensively reviewed recently [4,5,6] and are summarized in Figure 1.

### 1.2. AhR Ligands

Different exogenous AhR ligands have been identified including not only classic xenobiotic AhR agonists (such as 2,3,7,8-tetrachlorodibenzo-p-dioxin (TCDD) and polycyclic aromatic hydrocarbons (PAHs)) [7], but also bacterial pigments from Pseudomonas aeruginosa and Mycobacterium tuberculosis [8], and even allergens ([9] and unpublished data). Endogenous ligands include tryptophan metabolites like the 6-formylindolo[3,2-b]carbazole (FICZ), the kynurenines, ligands provided by commensal microbiota [10], dietary components derived from vegetables [11] and other metabolites such as bilirubin and arachidonic acid derivatives [12,13]. The AhR ligands discussed in this review are listed in Table 1. Recent data have shown that AhR is critical for a wide range of immune functions including the maintenance of innate and adaptive cell populations at mucosal barrier sites, and the control of inflammation at steady-state or during ongoing inflammatory responses, such as asthma.

## 2. AhR Is Expressed in Many Immune Cells Involved in Asthma Pathophysiology

**Figure 2:**
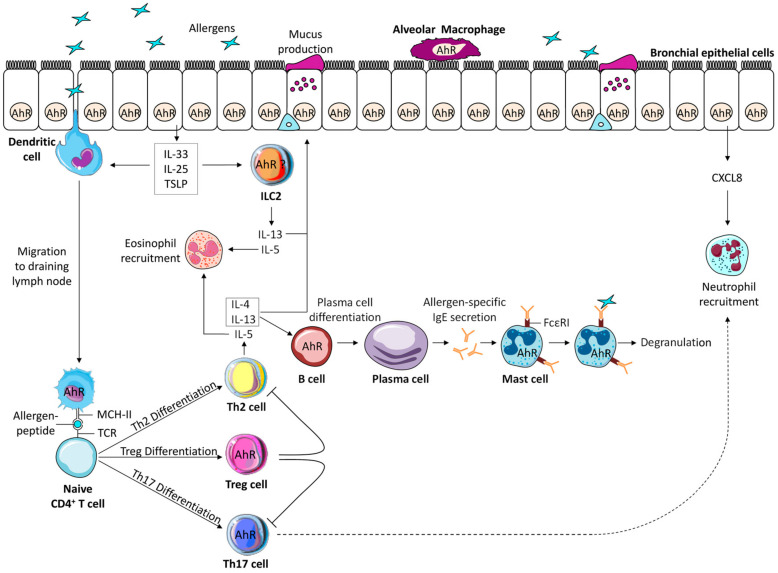
Aryl hydrocarbon receptor (AhR) expression in the different cells involved in the pathophysiology of asthma. The asymptomatic sensitization phase involves allergen presentation by antigen presenting cells to naive T cells leading to Th2 differentiation, production of interleukin (IL)-4 and IL-13 that activates B cells to differentiate into plasma cells producing allergen-specific IgE that bind to high affinity IgE receptors expressed by mast cells. Among these cells, macrophages, dendritic cells, and B cells express AhR. The effector phase of the asthmatic reaction following a second allergen contact, involves IgE dependent immediate release of mast cell mediators leading to bronchial smooth muscle cell contraction, activation of epithelial cells that release the pro-type 2 cytokines IL-33, Thymic Stromal Lymphopoietin (TSLP), IL-25, activation of innate lymphoid cells type 2 (ILC2) and Th2 cells and eosinophil recruitment. In some forms of asthma, Th17 cells are also activated leading to the recruitment of neutrophils, which are also attracted by the release of CXCL8 by epithelial cells. Epithelial cells, mast cells, Th17 cells and potentially ILC2 express AhR. Finally, an important negative regulator of the allergic reaction is represented by Treg that also express AhR. The co-activation of all these cells by AhR ligands can modify the outcome of the asthmatic reaction (see text for details).

Asthma is characterized by a bronchial inflammatory reaction and an abnormal bronchospastic response to allergens and irritants, in which several cellular components of the innate and adaptive immune system act together with epithelial cells to cause bronchial hyperreactivity. Many immune cell types involved in the asthmatic response express AhR (Figure 2), and their co-activation by AhR ligands can regulate the outcome of the reaction.

Among asthma phenotypes, the most common is the type 2 phenotype triggered by allergen stimulation [46,47]. The pathophysiology of the asthmatic reaction starts with an asymptomatic sensitization phase involving lung antigen-presenting cells, that while being exposed to environmental and endogenous AhR ligands, uptake and process the allergen. Then, allergen presentation by dendritic cells (DC) to naive T cells leads to the production of Th2 cytokines and to the activation of B cells that differentiate into allergen-specific IgE-producing plasma cells. These IgE antibodies bind to the high IgE affinity receptors expressed on mast cells and basophils awaiting a later allergen exposure. Both DC and macrophages express AhR, and recent data show that AhR acts upstream of DC by directing human monocyte fate towards DC rather than macrophages [48] suggesting that AhR stimulation may favour DC-dependent mechanisms.

A second allergen contact triggers the effector phase of the reaction. Crosslinking of the allergen to IgE bound to the high affinity IgE receptors expressed by mast cells and basophils induce the release of mediators (including preformed histamine, and synthesized prostaglandins and leukotrienes) leading to smooth muscle bronchoconstriction. In this context, it is of interest that human and murine mast cells constitutively express AhR, and that antigen-specific IgE crosslinking in the presence of a single dose of the AhR ligand FICZ boost mast cell degranulation whereas repeated exposure leads to inhibition of degranulation [16]. It was recently shown that part of FICZ effect was mediated by ORMLD3 (an asthma candidate gene) that induces sphingosine-1-phosphate metabolite and activates mast cell degranulation [49,50,51]. Altogether, these data suggest that AhR in mast cells may play a role in the immediate allergic reaction.

Allergen exposure also activates lung epithelial cells and their release of numerous pro Th2 cytokines such as interleukin (IL)-33, IL-25 and Thymic Stromal Lymphopoietin (TSLP), and chemokines such as C-C motif ligand (CCL)11 (eotaxin-1) and CCL17 (TARC). Epithelial cells constitutively express AhR, and as a cell at the interface with the environment, is central to the regulation of the asthmatic reaction. In human bronchial epithelial cells, while constitutively active unligated AhR suppresses the production of the chemokines CXCL8 and CCL5, AhR ligands alone induce the production of CXCL8 [52], underscoring the duality of AhR in inflammation, starting at the level of epithelial cells.

The release of the aforementioned mediators drives the development of the allergic inflammatory reaction, characterized by pulmonary recruitment of Th2 cells, eosinophils, airway hyperresponsiveness and mucus hyper-secretion [53,54]. Besides the classical adaptive Thelper (Th) responses, a novel actor of the asthmatic reaction belonging to the family of Innate lymphoid cells (ILC) [55], has been more recently uncovered. ILC are preferentially distributed at mucosal sites, do not express antigen receptors, but respond to cytokines in their environment. The cytokine pattern that they produce mirrors the classical T helper cell populations, with type 1 ILC1,type 2 ILC2 and type 3 ILC3 being the innate counterparts of respectively Th1, Th2 and Th17/Th22 cells. ILC2 produce IL-13 and IL-5 upon stimulation by IL-33, IL-25 or TSLP and contribute to clinical and experimental asthma, through induction of lung eosinophilia, mucus hyper-production and airway hyperresponsiveness [55]. Hitherto, lung ILC3 have been found only in some forms of experimental asthma, in particular associated with obesity [56,57]. Ahr is highly expressed in gut ILC2 and ILC3, and gain of function of *Ahr* results in ILC2 suppression but promotes ILC3 function [58,59,60,61], suggesting that Ahr favours Th17-type cytokine production by subtle counter regulation in ILC subsets at least at the gut level. Although several studies have shown that AhR ligands containing pollutants induce Th17-type responses, no study has yet explored their effects on lung ILC3.

Some forms of asthma exhibit a Th17 high profile that may contribute to their severity, whereas the induction of Treg is considered as beneficial. AhR expression varies in different T cell subsets, with levels being the highest in Th17 and Treg cells, and the lowest in Th1 and Th2 cells [62]. It was only in the 2000 s that AhR took a central place in the regulation of T cell biology. It was shown that AhR is upregulated in developing Th17 cells, and boosts the production of Il-17 and Il-22 [63,64,65] in mice and of IL-22 in humans [66,67]. Accordingly, *Ahr* deficient mice exhibit decreased Il-22 production by Th17 cells [65]. However, Ahr ligands may differentially impact Th17-mediated disease outcomes with FICZ favouring and TCDD inhibiting this profile [64,65]. Ahr expression is induced by signal transducer and activation of transcription 3 during Th17 differentiation [68], and it suppresses Stat5 and Stat1 signalling that interferes with Th17 generation allowing a positive feedback loop [63]. Collectively, Ahr appears to play a role in the early stages of Th17 differentiation, whereas the development of fully pathogenic effector Th17 cells requires other factors [69].

However, Forkhead box P3 (FOXP3)^+^ T regulatory cells (Treg) and IL-10 producing type 1 T regulatory cells (Tr1) cells have also been linked to AhR activation [70,71]. In contrast to FICZ that promotes Th17 differentiation, AhR ligands such as TCDD or kynurenine increase *Foxp3* expression through different mechanisms including epigenetic modifications of T cells and modulation of DC [15,72]. These epigenetic modifications of T cells, in particular DNA methylation, allows differentiation of different sub-populations and contributes to flexibility and plasticity among CD4+ T cell subsets [73]. In asthma patients, increased methylation of *FOXP3* in peripheral blood Treg cells has been associated with their functional impairment in an environment of high ambient air pollution levels compared to asthmatic individuals with low air pollution levels [74]. For Tr1 cells, it has been demonstrated that their Ahr expression is boosted by Il-27, a pleiotropic cytokine with diverse immune regulatory activities, through a mechanism driven by Stat3 [75]. This activation influences the transcriptional program of Tr1 cells leading to the production of the immunosuppressive cytokine Il-10. It is noteworthy that activin-A, a member of the transforming growth factor-β (TGF-β) superfamily, has been described to induce the transcription factors IRF4 and AhR in human CD4^+^ T cells, leading to the generation of Tr1-like cells able to suppress allergic responses in a model of humanized mice [76]. Interestingly, in established Th17 cells, Ahr can convert them into Il-10-producing Tr1 cells [77], a process that has been observed in allergic rhinitis [78]. Therefore, Ahr may be linked to plastic Th17 cells that require additional information to complete their differentiation into either fully pathogenic Th17 cells or regulatory Tr1 cells.

In addition, the development of a humoral response in asthma is fundamental for the sensitization process and development of the immediate bronchospastic reaction through IgE synthesis. AhR is expressed in B cells, and a previous study has shown that the AhR agonist [4-(3-chloro-phenyl)-pyrimidin-2-yl]-[4-trifluoromethyl-phenyl]-amine (VAF347) inhibits IgE synthesis by human B cells [37]. More recently, the role of Ahr in B cells has been revisited and shown to regulate B cell fate decisions by favouring memory B cells but repressing terminally differentiated antibody-secreting plasma cells in vivo [79]. Moreover, Ahr can promote Il-10-producing B regulatory cells (Breg) [80], in part through its activation by microbiota-derived butyrate [81]. Therefore, AhR is also involved in B cell regulation and may serve as a molecular rheostat to brake the effector response, possibly to facilitate optimal recall responses.

In summary, AhR regulates the innate and adaptive response at multiple levels with lineage-specific effects likely to differentially regulate the outcomes of the asthmatic reaction. These outcomes are reviewed in the following sections, and show both beneficial and deleterious effects in the asthma response either by the use of gene silencing, knock out mice or AhR antagonists.

## 3. AhR and Its Beneficial Effects in Experimental Asthma

### 3.1. AhR Activation is Modulated by epigenetic Mechanisms in Bronchial Epithelial Cells, And Prevents Airway Inflammation

Epigenetic variations have been associated with asthma and its severity in various cells and have evidenced an important role for DNA methylation in asthma pathogenesis [82,83]. For example, ten-eleven translocation 1 (*TET1*) promoter methylation has been associated with childhood asthma [84]. In vivo, house dust mite (HDM)-challenged *Tet1^-/-^* mice exhibit increased allergic airway inflammation. Lung transcriptomic analysis of these mice has revealed a downregulation of genes involved in Ahr signalling, including *Cyp1a1* and *Aldh1a1*, and an increase in interferon signalling compared to *Tet1^+/+^* mice. In human bronchial epithelial cells, activation of TET1 also differentially regulates interferon and AhR signalling pathways. Genes in these pathways are associated with changes in DNA methylation, predicted binding of transcriptional factors with relevant functions in their promoters, and the presence of histone marks generated by histone enzymes that are known to interact with *TET1*. Collectively, these data suggest that TET1 prevents allergic airway inflammation through inhibition of the interferon and activation of the AhR signalling pathways in airway epithelial cells, probably through epigenetic modifications [85].

### 3.2. AhR Activation in Dendritic Cells Blocks the Generation of Pro-Inflammatory T Cells

Antagonizing AhR in human progenitor cells induces their differentiation in DC able to activate pro-inflammatory cells [86] suggesting an anti-inflammatory role of AhR in DC. Accordingly, *Ahr*^-/-^ mice sensitized and challenged with ovalbumin (OVA) develop an increased inflammatory response in the lung compared with wild type (WT) controls, with greater numbers of inflammatory eosinophils and neutrophils, increased T-cell proliferation, production of Th2 cytokines, and higher levels of OVA-specific IgE and IgG1. Mechanistically, murine Ahr-deficient lung DC induce increased allergen-specific T cell priming, proliferation and levels of Th2 and Th17 cytokines, and are more highly activated than WT DC [17]. Furthermore, when murine pulmonary DC are incubated overnight with lipopolysaccharide (LPS) and OVA, the addition of 100 or 200 nM FICZ inhibits the proliferation of T cells demonstrating that Ahr is an important negative regulator of allergen-dependent T cell activation in vitro [17]. These data have been confirmed in an in vivo murine model of allergic asthma, where intraperitoneal injection of FICZ during sensitization to OVA reduces pulmonary eosinophilia and expression of Th2 cytokines. The mechanism involved is the inhibition by FICZ of Stat6, a transcription factor necessary for Th2 differentiation [18]. Other agonists of AhR have been reported to have similar effects. VAF347 is a low molecular weight compound, targeting only DC and B cells, initially screened for its capacity to inhibit IgE production in B lymphocytes. In vitro, VAF347 reduces the expression of IL-6, and of the stimulatory molecules CD86, and HLA-DR by DC, thereby blocking the generation of functional human Th cells. VAF347 is able to inhibit OVA-induced pulmonary allergic inflammation in vivo by blocking lung eosinophilia, serum IgE, and goblet cell hyperplasia [37]. These data and the fact that VAF347 similarly inhibits allergic lung inflammation in B cell-deficient mice suggest that its in vivo biological effects are mainly mediated through DC. Later on, AhR was identified as the pathway mediating the effects of VAF347. Accordingly, in a mouse model of allergic lung inflammation, VAF347-induced reduction in total IgE and Il-5 serum levels was abolished in *Ahr^−/−^* mice [38]. Therefore, the interaction of this compound with the AhR protein in DC is essential for the induction of a potent anti-inflammatory phenotype.

In summary, in these studies, early activation of AhR expressing DC either with FICZ or with VAF347 in the context of an allergic reaction is able to inhibit their ability to activate pro-inflammatory T cells.

### 3.3. AhR in Mesenchymal Stem Cells Shifts Macrophages towards An Anti-Inflammatory M2 Phenotype

Another beneficial effect of AhR has been shown at the level of mesenchymal stem cells (MSC). These cells are multipotent progenitor cells that express Ahr, suppress lung inflammation [87] and participate in tissue repair and remodelling by differentiating into a number of mature cell types. MSC control inflammation by balancing the polarization to pro-inflammatory M1 and alternatively activated anti-inflammatory M2 macrophages [88]. MSC have been involved in cockroach allergy. Cockroach allergen disturbs airway epithelial integrity through protease-activated receptor-2, which leads to increased penetration of allergens, directly activating epithelial cells and their production of cytokines and chemokines [89]. In a mouse model of cockroach allergen-induced allergic airway inflammation, an increased recruitment of MSC was observed in the airways, a process probably mediated through Tgf-β1 release, whereas in vitro, MSC inhibited cockroach allergen-induced CD4^+^ T cell cytokine secretion [90], suggesting a protective role of MSC in this model. In another study in the same model, Ahr was demonstrated to be activated by cockroach allergen in bone marrow-derived MSC [9] and to be necessary for MSC airway migration [9]. There was a protective role of Ahr in this model. Indeed, the deletion of Ahr, exacerbated cockroach allergen-induced lung inflammation with peribronchial inflammation, goblet cell hyperplasia and decreased CD4^+^CD25^+^Foxp3^+^ Treg and MSC recruitment [9].

Several AhR-dependent mechanisms have been involved in the suppressive effects of MSC. Ahr activation by TCDD in MSC upregulates the expression of inducible nitric oxide synthase (iNOS), which promotes the production of nitric oxide (NO), thus enhancing the inhibitory effect of MSC on lymphocyte proliferation [91]. Moreover, in the cockroach-induced asthma model, the AhR inhibitor CH223191 [42] antagonizes MSC-induced macrophage switch from a pro-inflammatory M1 towards an anti-inflammatory M2 phenotype [92], indicating a beneficial role for Ahr through M2 macrophages. In summary, the recruited MSC, activated through Ahr by environmental pollutants or cockroach allergen or both synergistically, release anti-inflammatory factors (e.g., iNOS and Tgf-β1) and modulate macrophage polarization that suppresses airway inflammation.

### 3.4. AhR Activation Suppresses Th2 Differentiation and Controls T Cell Activation

AhR plays an important role in T cell regulation. Among AhR exogenous ligands, TCDD is the most potent congener of chlorinated dioxins, a large class of environmental pollutants produced as a byproduct of various industrial processes. Many of the toxic effects resulting from TCDD exposure, including immunosuppression, are mediated by AhR. As stated in the previous section, TCDD has been mainly involved in the generation of Treg. In a non-eosinophilic model of OVA/LPS- sensitized mice, oral administration of TCDD decreased neutrophil recruitment, Th17 cytokine expression and airway hyperresponsiveness, but increased Il-10 production and CD4+CD25+ Tregs. In this model, DC maturation was not impaired [21] suggesting a direct effect of TCDD on T cells.

Besides TCDD, other AhR ligands can have inhibitory effects. The endogenous metabolite uremic toxin indoxyl 3-sulfate (I3S) is able to inhibit the differentiation of Th2 cells but not Th1 cells [14]. In a murine model of OVA-induced allergic asthma, intraperitoneal injections of I3S suppressed pulmonary eosinophilia, serum IgE level and lung Th2 cytokines. In this model, I3S decreased the frequency of Il-4-producing T cells in mediastinal lymph nodes and inhibited activation of Stat5 and Stat6 associated with Il-4-mediated Th2 differentiation. Accordingly, the AhR antagonist α-naphtoflavone suppressed the effects of I3S on Th2 differentiation [43], indicating that I3S regulates Th2 differentiation in an Ahr-dependent manner [14]. Therefore, I3S might be a potential therapeutic target in type 2 asthma.

**Figure 3:**
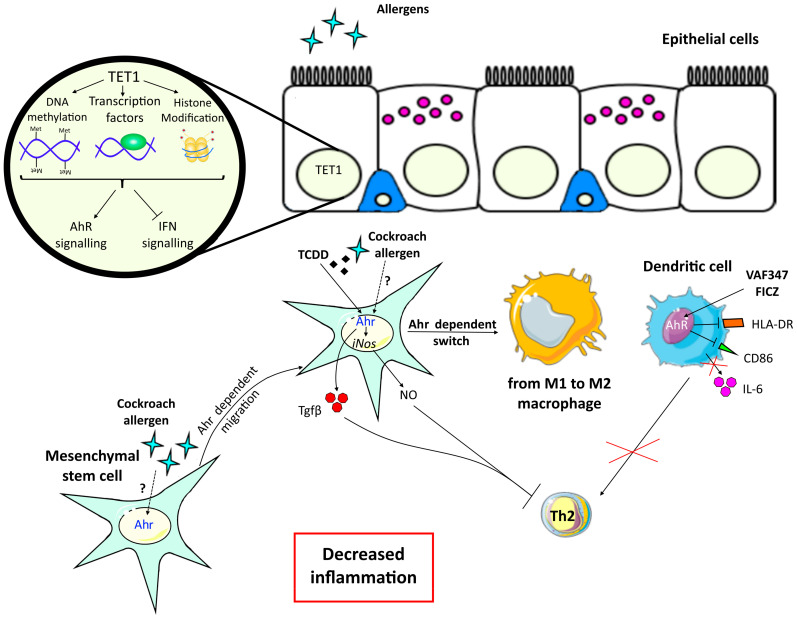
Beneficial effects of aryl hydrocarbon receptor (AhR) activation in asthma. Ten-Eleven Translocation 1 Gene Protein (TET1) is a demethylase that induces epigenetic modification and acts on DNA methylation, transcription factors binding or histone modifications. In epithelial cells, TET1 activation blocks interferon (IFN) signalling and induces AhR signaling, promoting an anti-inflammatory phenotype in asthma models. Mesenchymal stem cells (MSC) express Ahr that can be activated by cockroach allergen leading to their recruitment and migration to the airways. These cells then induce a switch from M1 to M2 anti-inflammatory macrophages. Ahr activation of MSC either by 2,3,7,8-tetrachlorodibenzo-p-dioxin (TCDD) or cockroach allergen upregulates the expression of inducible nitric oxide synthase (iNOS) and secretion of nitric oxide (NO) and Tgf-β leading to the inhibition of the T cell response. Activation of dendritic cells (DC) by AhR agonists formylindolo[3,2-b]carbazole (FICZ) or synthetic [4-(3-chloro-phenyl)-pyrimidin-2-yl]-[4-trifluoromethyl-phenyl]-amine (VAF347) impairs their ability to generate functional Th cells through a reduction of IL-6 secretion and of stimulatory molecules CD86 and HLA-DR expression. Altogether, AhR expression by epithelial cells, MSC and DC have a beneficial effect on asthma features.

The beneficial role of AhR on T cell activity modulation was also demonstrated in another study evaluating ozone, an air pollutant that causes respiratory inflammation and exacerbates asthma. Upon chronic ozone exposure in mice, Ahr is activated in the lung, and lipoxin A4, an anti-inflammatory molecule and AhR ligand [19] is released in broncho-alveolar lavage fluids [20]. In this model, *Ahr*-deficient mice exhibited an increase in cell recruitment, airway hyperreactivity, epithelial cell injury, inflammation, and fibrosis suggesting that Ahr regulates lung inflammation and epithelial damage after ozone exposure [20]. The expression of *Ahr* in T cells was necessary to control ozone-induced lung inflammatory cell infiltration, as shown in mice with a T cell-specific *Ahr* deletion. After 6 weeks of ozone exposure, IL-17 and IL-22 production were higher in the lung when the *Ahr* gene was missing, suggesting an impact of Ahr on the recruitment of lung Il-22-producing cells. For Il-17, Ahr acted directly on cell production and indirectly on cell recruitment. Lastly, Il-17- and Il-22- neutralizing antibodies attenuated lung inflammation in *Ahr^−/−^* and control mice [20]. Therefore, ozone exposure activates Ahr, which plays a beneficial role in controlling ozone-induced lung inflammation and airway hyperresponsiveness via the reduction of Th17 cytokine expression. The beneficial effects of AhR activation in asthma are summarized in Figure 3.

AhR ligand direct beneficial effects on T cells are shown in Table 2. Altogether, these data suggest that AhR can inhibit the asthmatic allergic reaction through either increased Treg or decreased Th2 cell activation according to the AhR ligand used. Of note, several natural compounds such as curcumin [39], flavonoids from dietary source [40] or compounds present in traditional medicine [41] have been shown to be AhR ligands and are currently proposed as anti-asthmatic agents. Nevertheless, the demonstration of their direct effect on asthma through Ahr signalling in animal models is still lacking.

## 4. AhR and Its Deleterious Effects in Experimental Asthma

### 4.1. AhR Increases Allergic Airway Inflammation by Activating Lung Epithelial Cells and Fibroblasts

Airway epithelial cells are able to modulate allergic airway inflammation by their ability to produce a variety of inflammatory mediators including the alarmins IL-33, IL-25 and TSLP, critical for driving type 2 inflammation in the lung tissue. In the human bronchial epithelial cell line BEAS2B, TSLP expression is induced by particle matter (PM) with a diameter <2.5 µm (PM_2.5_) in an AhR-dependent pathway [93]. These data are in agreement with the ability of AhR to bind alarmin promoters and activate their transcription [30]. Interestingly, in vitro co-exposure of human epithelial cell lines to the HDM allergen *Dermatophagoides farinae* (Der f) and Benzo(a)pyrene (B(a)P), increases the expression of TSLP and IL-33 compared to cells stimulated with one or the other compound, effect also observed in vivo [26,27]. Moreover, Wang et al. have shown that pre-incubation of epithelial cells with the AhR inhibitor CH223191 or transfection with a siRNA targeting the AhR decreases the expression of both cytokines, through effects on reactive oxygen species (ROS) production. Indeed, CH223191 inhibits ROS production and pre-treatment with the antioxidant N-acetyl-L-cysteine alleviates the secretion of TSLP and IL-33. In this study, results were confirmed in vivo in a mouse model of Der f 1 sensitization [26]. These studies suggest that AhR-dependent increased expression of alarmins by the lung epithelium may participate in the exacerbation of type 2 asthma by pollution.

Release of these epithelial alarmins can also activate ILC2 and while at the lung level, some AhR inducers such as diesel exhaust particles (DEP) and ozone [20] have been shown to enhance pulmonary ILC2 [31,94], the involvement of Ahr has not been evaluated. However, a recent study has shown that lung ILC2 that express the Il-33 receptor, produce IL-17 in response to in vivo papain challenge and that their production of Il-17, but not Il-5, is decreased in *Ahr* deficient mice [95] suggesting that Ahr in epithelial cells may also modulate Th17-type inflammation through ILC.

Another factor secreted by epithelial cells is mucin-containing mucus, which normally protects the airway from exogenous substances. Asthma is characterized by mucus hypersecretion by airway secretory club cells. These cells are very sensitive to AhR stimulation [22]. Wong et al. have reported that TCDD and urban dust increase the expression of the mucin gene *MUC5AC* via AhR signalling in a club cell line. Moreover, *in vivo*, a single intraperitoneal injection of TCDD increases Muc5ac, and the lung matrix metalloproteases (Mmp)12 and Mmp13, involved in tissue remodelling. Mice exhibiting an inhibitory mutation of Ahr (Ahr^NLS^, defective translocation to the nucleus) do not show such increased expression [22]. In HDM-sensitized mice, the addition of PM is also able to increase mucus production and *Muc5ac* expression in the lung, compared with HDM alone [32]. The mechanism has been further deciphered by Wang et al., who have shown in a mouse model of Der f 1-sensitized mice, that the additional B(a)P increased goblet cell hyperplasia occurs through an Ahr-dependent pathway [26].

Fibroblasts are important structural cells of the lung implicated in matrix protein production such as collagen or profibrotic cytokines such as TGF-β1. These cells are precursors for myofibroblasts involved in airway remodelling in asthma. The proliferation and the over-production of matrix mediators lead to a thickening of the airway wall and a decrease of the lumen. The WI-38 human lung fibroblast cell line constitutively expresses AhR. The treatment of these fibroblasts with cockroach allergen increases AhR and TGF-β1 expression. Interestingly, cockroach allergen-induced TGF-β1 is partly dependent on AhR activation since fibroblasts transfected with siRNA against AhR display lower expression of the cytokine compared to cells transfected with scrambled siRNA [23]. Moreover, costimulation of cockroach allergen-stimulated fibroblasts with the AhR agonist TCDD further enhances TGF-β1 expression, through an increase in latent TGF-β1 binding protein-1, which is inhibited by the AhR antagonist CH223191 [23]. In addition, cockroach allergen exposure of fibroblasts significantly increases the expression of alpha smooth muscle actin (α-SMA) also partly inhibited by *AhR* siRNA, suggesting that AhR induces fibroblast differentiation towards myofibroblasts, cells implicated in subepithelial fibrosis and airway remodelling in asthma. Su et al. have also shown that TCDD alone induces AhR-dependent α-SMA expression and migration of human HFL-1 fibroblasts [24]. Altogether, these results suggest that AhR may be a positive regulator of cockroach allergen-induced TGF-β1 signalling in human fibroblasts and that AhR exogenous ligands can enhance airway remodelling.

These data highlight the difference of effects of TCDD in asthma according to the cell type expressing AhR, beneficial when involving DC and Treg cells and deleterious for epithelial cells and fibroblasts.

### 4.2. AhR Activation Induces Dysregulation of Antigen Presenting Cells in Asthma

DC and macrophages are antigen-presenting cells that play a major role in priming adaptive immune responses. A major compound of PM_2.5_, the indeno[1,2,3-cd]pyrene, structurally similar to B(a)P, has been shown to increase type 2 inflammation in a mouse model of asthma. This exacerbation of the Th2 response was dependent on Ahr activation in DC, as shown in DC-specific *Ahr* knock out mice [28]. An exacerbating effect of vehicular ultrafine PM has also been observed in allergic airway inflammation in mice, by activation of the Ahr signalling cascade in DC, leading to elevated Il-17 responses in vivo as well as in vitro [33,34]. Mechanistically, costimulation of OVA-pulsed bone marrow derived-DC (BMDC) with PM_2.5_, induces Ahr-dependent increased activation of DC with increased expression of CD86 and MHC II, as well as of IL-1β compared to OVA stimulation alone [33]. Moreover, in co-cultures of OVA-specific DO11.10 T cell/OVA-pulsed DC with ultrafine particles (UFP), inhibition of Ahr induces a reduction in the secretion of Th2 and Th17 cytokines [34]. *In vivo*, exacerbation of the allergic response and Il-17 secretion by vehicular UFP, is mediated through a pathway involving Ahr-Jag1-Notch signalling in DC. More specifically, this induction is dependent on the activation of Ahr by PM-associated polycyclic aromatic hydrocarbons (PAH), which bind to the promoter of *Jag1* and induce its transcriptional activation. Then, Jag1 engages Notch receptors on allergen-specific T cells, leading to increased differentiation into Th2 and Th17 cells. Deletion of *Ahr* in lineage-specific CD11c^+^ cells confers protection against PM-mediated exacerbation of the allergic response and PM-mediated IL-17 increase, highlighting the key role of Ahr in CD11c^+^ cells, and in particular DC, in the polarization of the allergic reaction aggravated by UPF [34].

In addition to DC, lung macrophages have also been implicated in the uptake and response to PM [96]. Inflammatory stimuli, including allergens, alter the potency of lung macrophages as antigen-presenting cells, and exposure of macrophages to PM alters their function. Xia et al., have shown that the major targets of nano-sized particles in the mouse lung are macrophages [36]. In bone marrow derived-macrophages (BMDM), stimulation with PM_2.5_ induces Ahr-dependent increased expression of oxidative stress enzyme Heme-oxygenase 1 as well as of inflammatory cytokines Il-6 and Il-1β independently of OVA stimulation. Interestingly, co-exposure of BMDM to OVA and PM increases Ccxl2 in an Ahr-dependent manner [33]. This chemokine induced by IL-17 is a chemoattractant for neutrophils but also for airway smooth muscle cells [97]. B(a)P also triggers the expression of neutrophil attracting chemokines, as shown in human macrophages by the induction of CXCL8 through the binding of AhR to the XRE sequence on the *CXCL8* promoter. In mice, B(a)P intranasal instillation also increases Cxcl1 and Il-6 as well as neutrophil recruitment in broncho-alveolar lavages [98]. These observations support the implication of AhR dependent pollutant-activated macrophages in severe asthma associated with neutrophilia and airway remodelling. Among lung macrophages in steady-state conditions, 95% of the particles are internalized by alveolar macrophages (AM) while only 2 to 3% are internalized by interstitial macrophages (IM). In OVA-sensitized mice, this ratio is modified with an internalisation of 70% and 30% respectively [36]. As it was described previously for DC, the Ahr-Jag1-Notch pathway has been explored in macrophages and it has been shown that, in vitro, particularly in AM compared to IM or DC, UFP upregulate Jag1 expression, which is abolished by deletion of *Ahr* in myeloid lineage-specific CD11c^+^ cells. Similar results were obtained in vivo in OVA sensitized mice [36]. AM capacity to induce T cell polarization was analysed in vitro. Purified mouse AM stimulated with OVA alone induced increased proportions of T helper cells producing Il-17, Il-13 or Il-4 as well as 40% of Foxp3^+^ induced T regulatory cells (iTreg) and a small proportion of Ifn-γ^+^ Th1 cells. In UFP/OVA coexposure, Th2 and Th17 polarization was enhanced whereas iTreg and Th1 were inhibited. The UFP effect on OVA stimulated macrophages was completely abolished in *Jag1*^-/-^ cells [36]. These data suggest that as in DC, Ahr regulates Jag1 transcription in AM and increases Th2/Th17 cytokines, while iTreg are inhibited.

All these observations demonstrate an important role of AhR ligands from pollution in the dysregulation of antigen presenting cells in asthma leading to increased inflammation and severity of the disease. Here again, the same cells, i.e., DC, are involved in both beneficial and deleterious effects of AhR in allergic inflammation, yet involving different AhR ligands, suggesting that their affinity, and potentially their access to the transcriptional AhR targets, may modify the DC response.

### 4.3. AhR Ligands Increase Pathologic T Cell and Asthma Features

Antigen-presenting cell dysregulation by AhR-binding pollutants induces aberrant T cell responses particularly an increased Th2/Th17 pattern as described in the previous paragraph. In vitro studies have shown that AhR ligands can also act directly on T cells. Van Voorhis et al. have shown that ambient urban PM promotes Th17 polarization in vitro in an Ahr dependent way in mice [99]. These results have been further confirmed in mice and in humans. Indeed, a recent study has evaluated the in vitro effect of PM_2.5_ exposure on the differentiation of Th17 or Treg from either CD4^+^ T cells from mouse spleen or from human peripheral blood mononuclear cells. The authors found increased Th17 and decreased Treg differentiation dependent on AhR but independent of the presence of antigen-presenting cells [35]. In contrast, in a study using another AhR ligand, the coculture of mouse T cells with OVA- and PAH 4-nonylphenol -activated BMDC did not modulate Il-17 and Il-4, whereas Ifn-γ was largely inhibited compared to cocultures with BMDC activated with OVA alone leading to a dysregulation of the Th1/Th2 balance [29]. This discrepancy between studies may be explained by the presence or not of antigen-presenting cell secreting factors that modulate T cell differentiation, compared to less specific direct T cell activation and differentiation in the presence of anti-CD3/CD28 antibodies. Moreover, AhR activation in the two first studies was mediated by PM_2.5,_ which contain multiple PAH as potential ligands of AhR, whereas the last study used the single pure 4-nonylphenol PAH. Therefore, differentiation towards a particular Th profile may result from the integration of multiple signals absent in single PAH activation.

Similar contrasting results have been observed in vivo using different allergens. In Der f 1-sensitized mice, increased secretion of Th2, but not Th1 or Th17 cytokines, has been observed in the broncho-alveolar lavage of mice co-exposed with B(a)P, compared to Der f 1 alone. The inhibition of Ahr with the specific CH223191 inhibitor abolished this effect on Th2 cytokines [26]. Castañeda et al. have observed similar results using total HDM extract and ambient PM_2.5,_ with increases in Th2 but no differences in Th17 cytokines in the lung of co-exposed mice [32]. However, they did not explore the implication of Ahr. In another model of mice sensitized with OVA and the 4-nonylphenol or the indeno[1,2,3-cd]pyrene, Th2 cytokines were also increased in the lung of mice in an Ahr-dependent pathway [28,29]. In contrast, in a cockroach-sensitized mouse model, co-exposure with PM_2.5_ increased CD4^+^ Il-17^+^ T cells in lymph nodes while CD4^+^ FoxP3^+^ cells were decreased. Moreover, this co-exposure increased *Il-17*, *Rorγt* and *Il-4* expression in the lung whereas *Foxp3* was decreased and *Ifn-γ* was not modified showing a dysregulation in the balance between Th17 and Treg. This dysregulation was T cell/Ahr-dependent since mice deficient in *Ahr* in CD4^+^ cells displayed low *Il-17* and increased *Foxp3* levels in the lung [35]. To explain these findings a glycolysis related molecular mechanism was proposed. It has been previously shown that glycolysis regulates Th17 cell differentiation [100] whereas Treg are regulated by Tet methylcytosine dioxygenase-induced demethylation on the *Foxp3* promoter. In the cockroach model, the expression of the enzymes of the glycolytic pathway, as well as the glycolysis rate, were increased in PM_2.5_-stimulated Th17 cells [35]. This effect was mediated by the regulator of glycogenesis Hif-1α, as demonstrated by inhibition of Th17 differentiation in *Hif-1α* knockdown T cells. Hif-1α was also induced by PM_2.5_ in Treg, but the knockout had no effect on the decrease of Treg differentiation in vitro suggesting a different mechanism of regulation by Ahr. Indeed, in this same study, it was also demonstrated that Ahr bind to the promoter of *Got1,* the major transaminase catalysing the conversion of glutamate to a-ketoglutaric acid in T cells. Knockdown of *Got1* promoted the differentiation of Treg cells and rescued the inhibitory effect of PM_2.5_ on their differentiation. Finally, PM_2.5_ exposure upregulated *Got1* expression through Ahr, resulting in inhibition of Tet-2 activity and hypermethylation in the *Foxp3* locus, thereby impairing Treg differentiation [35]. Therefore, these data suggest that the different effects of the AhR ligands observed in vivo, are linked to epigenetic mechanisms that may vary according to the allergen used for the asthma model.

**Figure 4:**
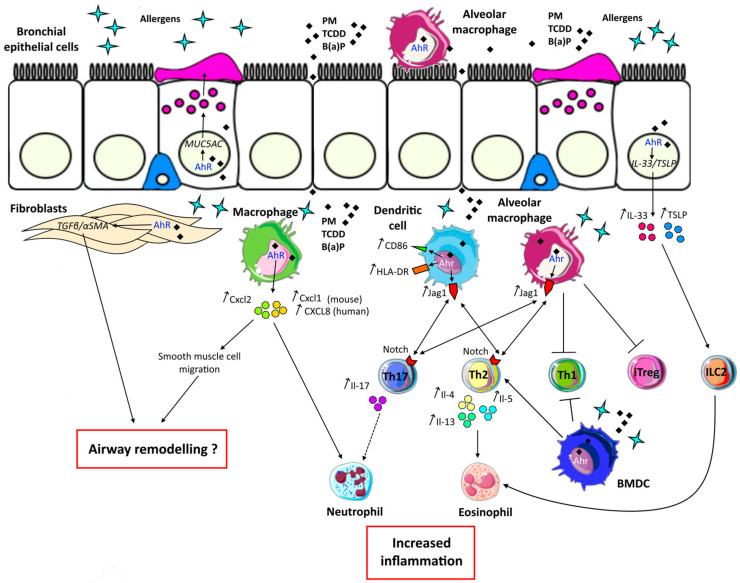
Deleterious effects of aryl hydrocarbon receptor (AhR) activation in asthma. Bronchial epithelial cells are the first barrier against environmental agents. In asthma, exposure to allergens and environmental AhR ligands such as particulate matter (PM), 2,3,7,8-tetrachlorodibenzo-p-dioxin (TCDD) or Benzo(a)pyrene (B(a)P) induces *MUC5AC* expression leading to mucus hypersecretion through AhR. Moreover, the alarmins interleukin (IL)-33 and Thymic Stromal Lymphopoietin (TSLP) are also induced through this pathway. These cytokines are able to activate innate lymphoid cells type 2 (ILC2) inducing eosinophil recruitment. Other structural cells implicated in asthma and expressing AhR are fibroblasts. Co-activation of these cells by an allergen and AhR ligands leads to *TGF*-β and *α-SMA* transcription, which are implicated in airway remodelling. Bone marrow derived-macrophages (BMDM) can also participate in airway remodelling through secretion of Cxcl2, a chemokine induced by Ahr activation and leading to smooth muscle cell migration. Moreover, increased secretion of Cxcl2 as well as Cxcl1 (or CXCL-8/IL-8 in humans) in response to allergen and AhR ligands costimulation leads to the recruitment of neutrophils. Neutrophil inflammation is associated with severe asthma and increased secretion of IL-17. The Th17 pathway is also induced by allergens and AhR ligands activated -dendritic cells and -alveolar macrophages through increased Jag1 expression. Furthermore, alveolar macrophages are implicated in Th1 and induced T regulatory cell (iTreg) inhibition through Ahr activation. In response to Ahr signalling, dendritic cells through increased expression of costimulatory molecule CD86 and HLA-DR, as well as alveolar macrophages participate in increased Thelper (Th)2 activation. Moreover, bone marrow derived-dendritic cells (BMDC) stimulated with an allergen and AhR ligands can amplify type 2 responses by inhibiting Th1 and activating Th2 cells. All these AhR-dependent signals result in increased Th17/neutrophilic and/or Th2/ILC2/eosinophilic inflammation, together with decreased Th1/Treg regulation.

The deleterious effects of AhR activation in asthma are summarized in Figure 4, except for AhR ligand direct effects on T cells, which are shown in Table 2.

From what has been discussed in this section we can conclude that many exogenous AhR ligands derived from pollutants, in particular PM and PAH, increase asthma features in animal models. In particular, inflammatory cell infiltration mostly eosinophils and/or neutrophils, inflammatory cytokine secretion, mucus production, airway hyperreactivity and IgE production are increased. Some differences are observed regarding the polarization profile, which may be related to the allergen and its composition, or to the AhR-expressing cell type initially activated. Regarding the differences in the effects of AhR on airway allergic inflammation, variations in ligands binding affinity and the cell types involved may explain the observed discrepancies.

## 5. AhR in Asthma Patients

Asthma patients display heterogeneous features including the type 2 eosinophilic and the non type 2 neutrophilic Th17 endotypes [13]. Surprisingly, few studies have evaluated AhR in samples from asthmatic patients. In peripheral mononuclear cells from uncontrolled allergic asthmatics, AhR and *IL-22* mRNA expression are increased compared with controlled asthma and healthy subjects, suggesting a relationship with asthma control [101]. A recent study has shown that bronchial epithelial cells derived from allergic asthmatic patients and stimulated with DEP produce Th2-inducing cytokines such as IL-33, IL-25 and TSLP, in an AhR-dependent manner. Moreover, severe asthmatic patients that exhibit high AhR nuclear translocation, have the highest levels of these type 2-inducing cytokines [30], indicating a link between AhR and severe allergic asthma. An increased expression of AhR has also been observed in fibroblasts from asthmatic patients compared to healthy donors [23], suggesting a potential participation in airway remodelling. In allergic asthmatic patients, it has been observed that the expression of IL-22 and IL-17, as well as the AhR target gene *CYP1A1,* in peripheral mononuclear cells is increased compared with healthy controls. However, stimulation with DEP increases AhR-dependent IL-22 production by CD4^+^ cells while decreasing IL-17 [66], implying that AhR differentially affects IL-17 and IL-22 production by mononuclear cells in humans as compared to mice. These variations could be related to species-specific effects of AhR agonists. Indeed, differences in ligand affinity have been observed between mouse and human AhR [102]. These affinity differences will have to be considered regarding the putative therapeutic applications in humans.

## 6. Conclusions

Taken together, all these data underpins the complexity of the AhR pathway, which leads to both beneficial and deleterious outcomes in asthma. The different outcomes can be explained by the ligand used and its affinity for AhR, the dose and the duration of AhR activation [25], by the cell subsets targeted and their plasticity, by the tissue involved and its local inflammatory environment, and by the accessibility of AhR transcriptional targets. For example, it was shown that microglial AhR exerts both pro-inflammatory and anti-inflammatory effects on the regulation of LPS-induced neuroinflammation, depending on the availability of external AhR ligands [103]. Such a mechanism could also occur in the lung but there are still many unknowns, which need to be solved before starting therapeutic manipulation of the receptor in a cell- or lung-specific manner. However, it is noteworthy that for the skin, drugs targeting AhR have been developed for the treatment of atopic dermatitis, an allergic disease characterized by a type 2 cytokine profile and recently described as elicited by pollutants through AhR [104]. Recent clinical trials using Tapinarof cream, an AhR activator, have shown encouraging preliminary results in atopic dermatitis [44,45] suggesting that AhR may represent a therapeutic target in allergic diseases.

## Figures and Tables

**Table 1:** List of discussed AhR ligands.

Compound	Class	Origin	Immune Mechanism in Asthma	Ref
I3S ^1^	Tryptophan metabolite	Uremic toxin from microbiota and host metabolism	Suppression of Th2 differentiation	[14]
Kynurenine	Tryptophan metabolite	Host metabolism	Induction of Treg generation	[15]
FICZ ^2^	Tryptophan derivative	Oxidation product of tryptophan (skin and microbiota metabolism)	Endogenous AhR agonist;Inhibition of Th2 differentiation; Th17 differentiation;Mast cell degranulation	[16,17,18]
Lipoxin A4	Arachidonic acid derivative	Host metabolism (immune cells)	Global reduction of inflammation	[19,20]
TCDD ^3^	Chlorinated dioxin	Released from industrial process	Depending on the model decreased Th17 cytokines and neutrophils with Treg induction or increased mucus production and profibrotic molecules	[21,22,23,24,25]
	Benzo(a)pyrene (B(a)P)	Pollutant	Mucus secretion, epithelial, macrophage, T cell activation: synergistic effect with allergen co-exposure	[26,27]
	Indeno[1,2,3-cd]pyrene	Pollutant	Dendritic cell activation: synergistic effect with allergen co-exposure	[28,29]
	4-nonylphenol	Pollutant		
Complex PAH ^4^ mix	Diesel exhaust particles (DEP)	Pollutant	Mucus secretion, epithelial, ILC2, T cell and dendritic cell activation: synergistic effect with allergen co-exposure	[30,31,32,33,34,35]
	Adsorbed on particle matters < 2.5 µm (PM2.5)	Pollutant		
	Adsorbed on ultrafine particles (UFP)	Pollutant	Dendritic cell, macrophage activation	[34,36]
VAF347 ^5^		Synthetic	Blockade of B and DC function	[37,38]
Flavonoïds	Polyphenolic metabolites	Vegetables (tea, garlic, berries, broccoli)	Immune mechanism unknown - used in traditional medicine as anti-asthmatic agents	[39,40,41]
Khellin & visnagin	Furanochromone derivative	Fruit of *Ammi visnaga*		
Curcumin	Diferuloyl methane	*Curcuma longa*		
CH223191 ^6^		Synthetic	AhR antagonist	[23,26,42]
α-naphtoflavone	Synthetic flavonoid	Synthetic	AhR antagonist	[43]
Tapinarof ^7^		Natural bacterial product	AhR agonist	[44,45]

^1^ Indoxyl 3-sulfate; ^2^ 6-formylindolo[3,2-b]carbazole; ^3^ 2, 3, 7, 8-tetrachlorodibenzo-p-dioxin. ^4^ polycyclic aromatic hydrocarbons; ^5^ [4-(3-chloro-phenyl)-pyrimidin-2-yl]-[4-trifluoromethyl-phenyl]-amine; ^6^ 1-Methyl-N-[2-methyl-4-[2-(2-methylphenyl)diazenyl]phenyl-1H-pyrazole-5-carboxamide; ^7^ GSK2894512.

**Table 2:** Direct Effects of AhR ligands on T cells in experimental asthma models.

Experimental Model	AhR Ligand	T Cell Response	Ref
Non-eosinophilic asthma model	2, 3, 7, 8-tetrachlorodibenzo-p-dioxin (TCDD)	Treg induction and inhibition of Th17 activation.	[21]
Ozone induced airway inflammation	Lipoxin A4	Inhibition of Th17/22 response	[20]
Ovalbumin-induced allergic asthma	Indoxyl 3-sulfate (I3S)	Inhibition of Th2 differentiation	[14]
	4-nonylphenol	Increased secretion of Th2 cytokines	[28,29]
	Indeno[1,2,3-cd]pyrene		
House Dust Mite-induced allergic asthma	Benzo(a)pyrene (B(a)P) or particle matter 2.5µm (PM_2.5_)	Increased secretion of Th2 but not Th1 or Th17 cytokines	[26,32]
Cockroach-sensitized mouse model	PM_2.5_	Increased Th17 cells and decreased Foxp3 regulatory T cells	[35]

## Abbreviations

AhR	Aryl hydrocarbon Receptor
AM	Alveolar macrophages
BMDC	Bone marrow-derived dendritic cells
BMDM	Bone marrow-derived macrophages
Breg	B regulatory cells
CCL	(C-C motif) ligand
DC	Dendritic cells
DEP	Diesel exhaust particles
Der f	Dermatophagoides farinae
FICZ	6-Formylindolo[3,2-b]carbazole
Foxp3	Forkhead box P3
HDM	House dust mite
iTreg	Induced T regulatory cells
IL-	Interleukin
ILC	Innate lymphoid cells
IM	Interstitial macrophages
IRF4	Interferon regulatory factor 4
LPS	Lipopolysaccharide
MMP	Matrix metalloproteinase
MSC	Mesenchymal stem cells
NO	Nitric oxide
OVA	Ovalbumin
PAH	Poly aromatic hydrocarbons
PM	Particle matters
ROS	Reactive oxygen species
SMA	Smooth muscle actin
STAT	Signal transducer and activator of transcription
TET	Ten-eleven translocation
TGF-β	Transforming growth factor-β
Th	T helper
Treg	T regulatory cells
Tr1	Type 1 T regulatory cells
TSLP	Thymic stromal lymphopoietin
UFP	Ultrafine particles
TCDD	2,3,7,8-tetrachlorodi-p-dioxin
WT	Wild type
